# Nemaline Myopathy With a Compound Heterozygous Mutation: A Case Report

**DOI:** 10.7759/cureus.63828

**Published:** 2024-07-04

**Authors:** Shiji Chalipat, Shruti Talewad, Aryan Gupta, Mridu Bahal, Shailaja V Mane

**Affiliations:** 1 Pediatrics, Dr. D. Y. Patil Medical College, Hospital and Research Centre, Dr. D. Y. Patil Vidyapeeth (Deemed to be University), Pune, IND

**Keywords:** nemaline myopathy, congenital myopathy, nemaline rods, heterozygous mutation, clinical exome sequencing

## Abstract

A class of genetically based congenital myopathies known as nemaline myopathies is defined by the development of nemaline rods within muscle fibers. We present a case involving an eight-year-old boy who presented with a history of delayed motor development, proximal muscle weakness, and neck flexor weakness. Muscle enzymes were normal, and electrophysiological studies revealed a myopathic pattern. Nemaline myopathy (NM) was diagnosed with the help of clinical exome sequencing, which showed a compound heterozygous mutation with a novel variant in the nebulin (NEB) gene.

## Introduction

Nemaline myopathy (NM) is a genetically determined congenital myopathy marked by the development of nemaline rods within muscle fibers that stain red with modified Gomori’s trichrome staining [1]. It has an incidence of 1 in 50,000 live births [2]. Common presentations include hypotonia, proximal muscle weakness, facial weakness, and skeletal deformities. Congenital, childhood, and adult forms are described. Cardiomyopathy can sometimes be associated due to a close structural resemblance between cardiac and striated muscles. Mutations associated with the disease have been described in at least 12 genes to date, with nebulin (NEB) and skeletal muscle (ACTA1) being the most common [1]. Here, we describe a case of NM with a novel genetic mutation.

## Case presentation

An eight-year-old male child presented with a history of delayed motor development and proximal weakness. He was born to unrelated parents at term gestation by normal vaginal delivery, with a birth weight of 2.5 kg and an uneventful perinatal period. The mother did not report any history of decreased fetal movements or polyhydramnios during the antenatal period. The postnatal period was uneventful, with no breathing or feeding difficulties. All motor milestones were attained late, and currently, he can only stand and walk with support. He could not self-raise to a sitting or standing posture. The rest of the milestones were achieved at the appropriate age. No diurnal variation of weakness was reported, and extraocular and bulbar muscles were spared. He had no sensory involvement or recurrent non-healing trophic ulcers.

**Figure 1:**
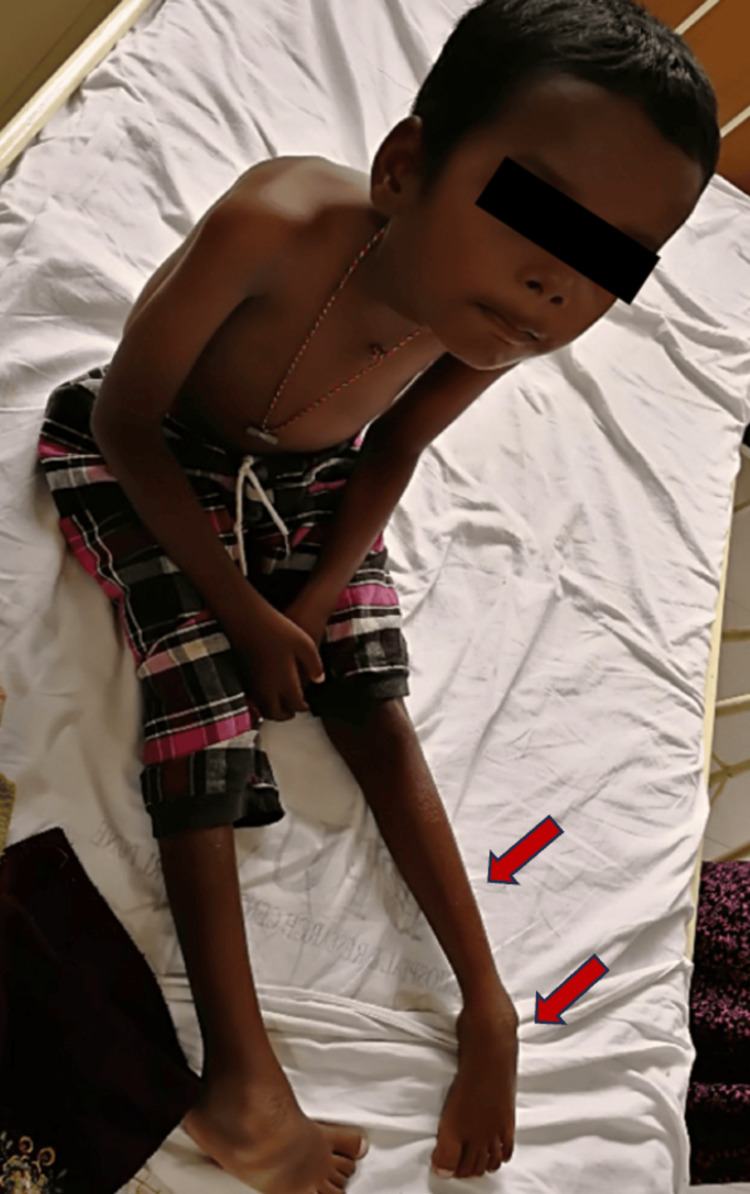
Atrophy of lower limb muscles and contractures.

**Figure 2:**
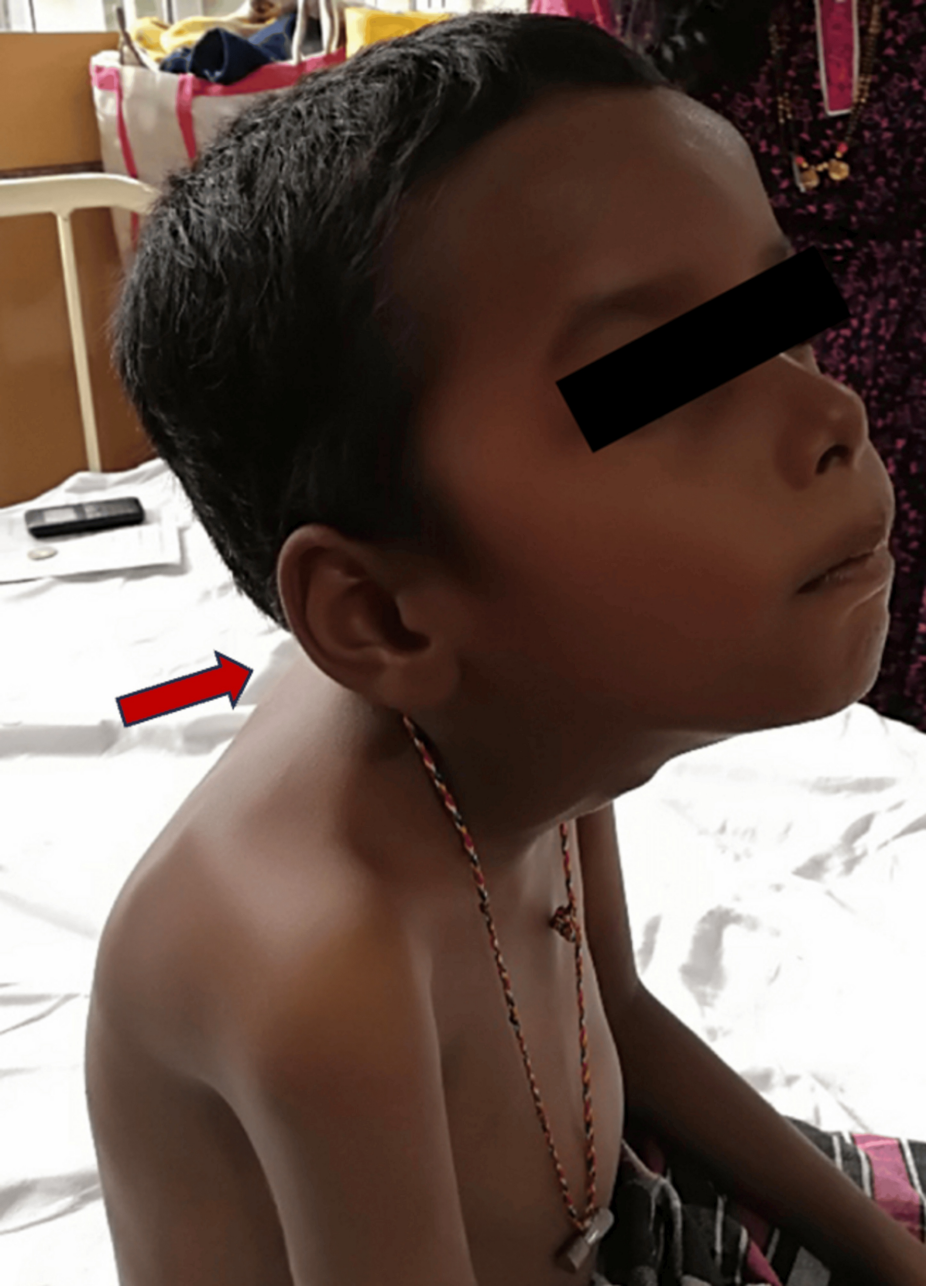
Neck flexor weakness.

Upon examination, the child had an elongated, expressionless face and a high arched palate. His anthropometric parameters showed normocephaly (head circumference (HC): 51 cm), and the child was undernourished. Higher mental functions and cranial nerve examinations were normal. Generalized muscle wasting (Figure 1) was noted, with a proximal more than distal distribution. Hypotonia was present in all four limbs. Power examination showed proximal weakness (grade 3) more than distal (grade 4) with depressed deep tendon reflexes. Neck flexors were weak (Figure 2), with affected muscles of facial expression. Extraocular muscles and bulbar muscles were spared. No fasciculations or muscle hypertrophy was seen. Fixed contractures were noted over bilateral ankle joints with plantar flexion and inversion deformity. The rest of the neurological and systemic examinations were normal.

Baseline blood investigations were normal. The CPK level was 45 (10-120 mcg/l), and vitamin D3 levels were 14 ng/ml. A nerve conduction study (NCV) was conducted, demonstrating a decrease in compound muscle action potential (CMAP) amplitude in the motor conduction study with normal conduction velocity. Sensory nerve conductions were normal. Electromyography (EMG) of sampled muscles showed a myopathic pattern with small amplitude, short duration, polyphasic motor unit action potentials (MUAPs) with early recruitment. In view of the normal muscle enzymes and electrophysiological studies consistent with a myopathic pattern, congenital myopathy was considered, and genetic testing was organized. The cardiac evaluation was normal, with a normal ECG and echocardiography. A pulmonary function test was also conducted and was normal. Clinical exome sequencing revealed a novel compound heterozygous mutation associated with NM. The reported mutation was a heterozygous eight base pair deletion at the exon-intron boundary of exon 113 and intron 112 of the NEB gene (c.17737-2_17742delAGTATCTC), which was a likely pathogenic variant. Another heterozygous missense variant (c.6623C>T) in exon 50 of the NEB gene resulted in the substitution of proline for leucine at codon 2208 (p.Pro2208Leu). Parental testing was advised but could not be done due to non-affordability. The child was started on regular rehabilitation therapy, including muscle strengthening and range of motion exercises, along with nutritional supplements and respiratory care.

## Discussion

NMs are a group of genetically determined congenital myopathies which can be inherited as autosomal dominant and recessive forms. Shy GM et al. recommended naming the condition "nemaline myopathy" from the Greek word for thread, "nema." [3]. It can result from mutations in 12 different genes, the two most prevalent of which are those encoding ACTA1 and NEB [1]. Neonatal NM has been documented in autosomal recessive instances caused by mutations in NEB [4,5] and tropomyosin [6], as well as dominantly inherited actin mutations [6]. Childhood-onset illness has been found with both dominant tropomyosin and actin mutations.

To aid in the identification of new genes, a clinical categorization was established during an ENMC workshop in 1999 [7]. Since then, more data have been collected, demonstrating that genotype-phenotype relationships are frequently absent or very weak. Therefore, a revised classification was proposed, which includes (a) Severe NM with severe contractures and respiratory involvement at birth; (b) Congenital NM with perinatal onset and delayed development; (c) Mild NM with childhood or juvenile onset; (d) Recessive TNNT1 (Amish) NM; (e) Childhood-onset NM with slowness of movements and core-rod histology [8].

Our patient presented with an uneventful perinatal period and delayed attainment of motor milestones, with early onset of slowly progressive proximal muscle weakness, classified under congenital NM. The typical presentation is slowly progressive or non-progressive weakness in proximal muscles, with later involvement of distal muscles. Neck flexor weakness, and facial, bulbar, and respiratory muscle involvement are also seen. Children with NM usually have hypotonia, a dolichocephalic head, and dysmorphic findings such as a high arched or cleft palate, retrognathia, and a long, expressionless face. Our child had neck flexor and facial muscle weakness, along with the above-described typical myopathic facial features. When compared to muscular dystrophies, it has a normal or slightly raised level of creatine kinase. EMG results might be normal or exhibit general myopathic features. Our patient had EMG findings consistent with a myopathic process. Although cardiac involvement in NM patients is uncommon, it has been seen in a small number of individuals with mutations in ACTA1, MYPN, or MYO18B [9,10].

A definitive diagnosis can be made through muscle biopsy or genetic testing. Muscle biopsy in NM shows the formation of nemaline rods in muscle histochemistry, which consist of excessive Z-band material and stain dark red on modified Gomori's trichrome staining. In our case, a muscle biopsy could not be performed as the parents were not willing. Clinical exome sequencing revealed a compound heterozygous mutation in the NEB gene. The NEB gene comprises 183 exons that encode a theoretical 26 kb full-length mRNA. A large number of pathogenic variants have been identified in NEB, but no definite phenotype-genotype correlations have been found. All pathogenic variants have been recessively inherited and are mostly compound heterozygous. A missense mutation combined with a more disruptive nonsense mutation results in a typical NM [11,12]. Both mutations noted in this child are not reported in the literature. Due to non-affordability, we could not proceed with parental genetic testing to confirm heterozygosity in them.

## Conclusions

For people with NM, there is currently no curative therapy. Treatment is mainly supportive and symptomatic. Tyrosine treatment has been tried, but there is no definitive clinical evidence supporting its effectiveness. Maintaining muscular strength and mobility through frequent physiotherapy is essential for managing the condition and ensuring independence in everyday activities. Regular monitoring of respiratory function and timely management of orthopedic problems like contractures and scoliosis is needed. Death usually occurs due to respiratory insufficiency. With this case report, we want to sensitize clinicians to the common congenital myopathy and its genetic basis of compound heterozygosity in the NEB gene.
